# Students’ attitudes and experiences toward mental health support services in Ireland: A qualitative study

**DOI:** 10.1371/journal.pone.0329905

**Published:** 2025-08-21

**Authors:** Olugbenga Oti, Sarah Foley, Ian Pitt

**Affiliations:** 1 Insight Centre for Data Analytics, School of Computer Science and IT, University College Cork, Cork, Ireland; 2 School of Applied Psychology, University College Cork, Cork, Ireland; Dublin City University, IRELAND

## Abstract

**Background:** There is a high prevalence of mental health difficulties among students in higher education. Barriers to seeking mental health support are well documented in this population. However, there is very little research on students’ experiences of accessing mental health support services in Ireland. This study aims to: 1) explore students’ experiences of accessing mental health support services in Ireland, and 2) investigate barriers to the use and continuous utilisation of these services.

**Methods:** We distributed an anonymous online survey to students at University College Cork through mailing lists and posters. The results presented in this study are a thematic analysis of the open-ended responses describing experiences with mental health support services before and during the COVID-19 pandemic.

**Results:** This analysis produced three main themes encompassing students’ experience seeking support. They include: 1) barriers to service use and engagement, 2) impact of COVID-19 restrictions on service use, and 3) barriers to continued use of support. These themes summarise barriers encountered by students throughout their help-seeking journeys. The process of seeking support was not straightforward, and students often encountered barriers that prevented or delayed their use of services.

**Conclusion:** There is a need to improve mental health literacy in this population, specifically, teaching students how to access support and what to expect from services. In addition, we recommend that mental health support services improve the process of gaining entrance to a service, by making it better suited to the student population.

## Introduction

In the past few decades, there has been an increase in depression [1,2] and suicidality [1] among college students. In 2018, the World Health Organisation surveyed first-year college students across 8 countries and 19 colleges. They found that one in three students screened positive for a mood, anxiety or substance use disorder [3]. These studies emphasise the importance of early treatment and prevention of mental health difficulties in this population [1,3,4].

Prior to the COVID-19 pandemic, research has documented barriers to accessing mental health support in the young adult population. For example, Mughal *et al.* [5] conducted a qualitative study exploring young adults’ experiences of care and access to care for self-harm in the UK. Barriers reported include dissatisfaction with public services, fear of loss of confidentiality, and prioritization of medication over therapy [5]. Participants were reluctant to use services because they felt they were not in the right mindset, they had heard negative stories about services and they were afraid of disclosing their difficulties [5]. Similarly, a mixed methods study explored students’ experiences of mental health support services in the UK [6]. Approximately one-third of participants rated the NHS mental health services as poor or very poor. Participants noted the need for shorter waiting times, the need for alternative treatments (if the current one was not working), more regular/longer appointments and referrals for non-crisis cases [6]. These studies provide context to barriers young adults face while accessing mental health support in the UK.

In June 2023, the European Union conducted a quantitative survey among the general population in Ireland and across member states. Findings revealed that Ireland was the hardest place in the EU to access mental health services [7]. Barriers to accessing support include long waiting lists, delays in treatment/diagnosis, cost and lack of awareness of suitable services [7]. Similarly, prior to the pandemic, the Union of Students in Ireland conducted a mixed methods survey among students in third-level institutions in Ireland. Their findings revealed that students faced structural barriers such as waiting lists, a limit on the number of counselling sessions, the inability to change counsellors, and a lack of flexibility around appointments [8]. In addition, students reported negative experiences with professional services which affected their engagement with services, for example, a prioritisation of medication over therapy [8].

Our research study was conducted during the COVID-19 pandemic. COVID-19 measures implemented in Ireland included travel restrictions (e.g. not more than 2km or 5km from home), closure of schools and third-level institutions and social distancing [9,10]. In addition, non-essential (non-COVID-19) services including General Practitioners (GPs) [10] and mental health support services [11] were shut down, meaning that many services were provided remotely [10,12]. A survey by the Irish College of General Practitioners revealed a shift to telemedicine consultations from 10.5% to 57% before and during the COVID-19 pandemic, respectively [13]. In addition, youth mental health services in Ireland recorded heightened levels of anxiety, isolation, loneliness and depression [11]. Within the first six months of the pandemic, Spunout, a youth mental health service, recorded a 100% increase in those seeking support for anxiety and depression [10].

Despite the increased mental health needs during the COVID-19 pandemic, reluctance to seek support remained present in this population [14].

A qualitative study conducted in the UK revealed that young adults did not use the National Health Service because they did not want to overburden the service [14]. YoungMinds, a UK based organisation, conducted mixed methods surveys during the COVID-19 pandemic between 2020 and 2021 [15–17]. They found that the pandemic had a negative impact on the mental health of approximately 82.5% of young adults [15,16] and about 28.5% of young adults had lost access to support due to the pandemic [15,16]. Of those receiving support, 73% received virtual support while only 8% received face-to-face support [17]. During this period, young adults reported several challenges in accessing support including lack of privacy at home to attend remote appointments, cancellation of face-to-face support and peer support groups, cancellations due to COVID-19 symptoms, and shortened appointments [15–17]. Finally, some young adults felt that virtual appointments were less effective than face-to-face support, stating that it was difficult to discuss their issues over phone or video calls. [15–17].

To our knowledge, only the Union of Students Survey reports on students’ experiences accessing mental health support services in Ireland. Nonetheless, this report fails to consider the entire help-seeking journey of students and does not reflect the experiences of the current generation of students. An understanding of students’ help-seeking journeys is essential for identifying gaps in the current provision of mental health support, ensuring that students are well-supported in their journeys to accessing support. Therefore, the objectives of this study are 1) to extend the findings of the Union of Students’ Survey by exploring up-to-date perspectives of students who have been through the process of accessing mental health support services in Ireland and 2) to investigate the barriers to access and continuous utilisation of mental health support services among college students in Ireland. It should be noted that these experiences encompass the periods before and during the COVID-19 pandemic.

In the present study, when quoting references, the terms young adults and students are used interchangeably. This is because participants in these studies include students in third-level education among other participants. In addition, we define access as the “actual use of personal health services and everything that facilitates or impedes their use” [18, p. 3]. In this definition, we acknowledge that barriers emerge at various stages of the help-seeking process and that barriers still occur when an individual has begun using services.

## Materials and methods

### Study design

We conducted an anonymous online survey to examine the experiences of students who had successfully/unsuccessfully accessed mental health support services within and outside their university. The survey aimed to capture: 1) demographic information, 2) help-seeking behaviours and experiences during and prior to the COVID-19 pandemic, 3) experiences providing support to peers, and 4) self-rated mental health and wellbeing over the past two weeks. Findings related to peer support and experiences with technological mental health support are under review or reported elsewhere, respectively. In the present study, participants were asked (Yes/No) if they had experienced significant problems in the past year (March 2020 - April 2021) and felt they would have benefited from professional help (e.g., counsellors, psychiatrists, GPs). The rest of this section of the survey was not relevant to those who responded “no”, and they were excluded from the dataset and the subsequent analysis. Those who responded “Yes” were asked what forms of support they had accessed during this period. Further, they were asked to give open-ended responses detailing their experiences with the support they received during the COVID-19 pandemic. In particular, they were asked to elaborate on their experience reaching out for these supports, how helpful/unhelpful the support was, and if they would continue using that form of support.

Furthermore, participants were asked (Yes/No) if they had ever used the UCC counselling service, other on-campus services and external services, i.e., services outside the university to receive mental health support. The rest of this section of the survey was not relevant to those who responded “no”, and they were excluded from the dataset and the subsequent analysis. Those who responded “Yes” were asked to give open-ended responses detailing their experiences using these services. In particular, they were asked to elaborate on the process of getting to know about a service, getting an appointment, the benefits of the service and the shortcomings of the service.

Finally, respondents were asked if they had any thoughts about the kind of mental health resources or support that students need, how mental health supports might be improved for students, or any other comments they would like to add. Personal experiences detailing the use of mental health support services were included in our analysis. The survey was developed in Google Forms. It was informed by previous young adults’ mental health surveys in Ireland [8,19–21]. It was also reviewed by academic staff in the School of Applied Psychology at University College Cork (UCC), who have experience in psychological instrument design.

Study advertisement and data collection began on February 10, 2021, and concluded on April 30, 2021.

### Study advertisement

The study was advertised on the UCC campus via posters and Twitter (now known as X), both of which included a link to the survey. Within UCC, posters were placed in strategic locations, for example, campus libraries and the student union building. In addition, the survey was sent to all students (undergraduate and postgraduate) in the university through the central mailing list. Further, requests were made to reach postgraduate students via the Postgraduate Research (PGR) mailing list and departmental mailing lists. We received confirmation that the survey was advertised to postgraduate students via the PGR and the College of Medicine and Health graduate school mailing lists.

### Informed consent

Participants provided informed consent digitally through the online survey, and they gave consent for the information provided to be used in research publications. The consent was provided by selecting “Yes” to the question “Do you consent to participate in this study?”.

### Ethical approval

Ethical approval was obtained from the University College Cork (UCC) Social Research Ethics Committee (SREC) under case number Log 2020-196A1.

### Inclusion and exclusion criteria

Participants were included in the survey if they were students at University College Cork and aged 18 years or older. Participants who were not current students at University College Cork or were under 18 years old were excluded from the survey.

### Analysis

**Fig 1:**
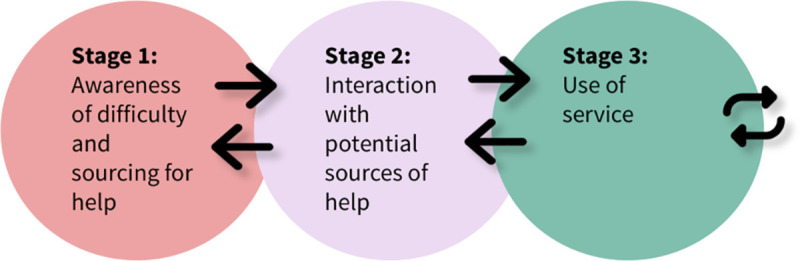
Model of help-seeking applied in this work (This image was created by OO).

The open-ended responses were analysed using the thematic analysis methodology of Braun and Clarke [22], now called reflexive thematic analysis [23]. The authors [22] posit six phases of analyses including 1) familiarisation with the data, 2) generating initial codes, 3) searching for themes, 4) reviewing themes, 5) defining and naming themes and 6) producing a report. The results were analysed in NVivo version 1.5.1. The first and second authors studied the data to gain familiarity with its content. Preliminary analysis of the data by the first and second authors further built familiarisation and immersion in the data. The first author conducted an inductive coding process, with codes bearing close similarity to the content of the data. We sought to examine students’ perspectives on seeking mental health support and uncover the barriers to access and continuous utilization of support services. Our search for themes was underpinned by the model of help-seeking, which posits that the experiences described by participants are on a timeline from their awareness of their need for support to their use of services. The model of help-seeking presented in this work is informed by our data, Rickwood’s model of help-seeking [24] and Andersen’s health service delivery framework [25]. A rationale for the model is included in the appendix. This model (see Fig 1) posits three stages in the help-seeking process, namely, “Awareness of problems and sourcing for help”, “Interacting with potential sources” and “Use of services”.

In the first stage, “Awareness of problems and sourcing for help”, students have an awareness that they have a problem with which they require some help. In addition, they are considering where to go to obtain mental health support. In the second stage, which is “Interacting with potential sources”, students actively interact with potential mental health support services to select one or more services they can use. In the third stage which is “Use of services”, students are actively using services to obtain mental health support. The arrows (see Fig 1) in each direction represent the progression or regression between stages, depending on barriers and facilitators. In the last stage, the feedback loop represents the distinction between initial entry and continued use of a service.

The themes presented in this paper are latent as they are grounded in the model of help-seeking applied in this work. The first author developed the initial themes. Discussions between the first and second authors led to further refinement of the themes. Finally, the first author wrote the report, which was refined with input from the second and third authors.

## Results

### Participant characteristics

There were a total of 475 survey respondents. The application of exclusion criteria led to the removal of 11 responses. In addition, 12 duplicate responses were removed.

A total of 452 respondents completed the survey. Most were female, aged between 18 and 22 years, and nationals of the Republic of Ireland (see Table 1 for the full demographic information).

**Table 1:** Demographic information.

Demographic Variables	Category	Count (n)	Percentage (%)
**Gender**	Female	313	69.2
	Male	126	27.9
	Non-binary	9	2.0
**Age**	18-22	271	60.0
	23 and above	181	40.0
**Place of Origin**	Republic of Ireland	352	77.9
	European Union	40	8.8
	North America	18	4.0
	Africa	18	4.0
	Asia	14	3.1
	United Kingdom	7	1.5
	South America	2	0.4
**Academic Discipline**	Arts, Celtic and Social Sciences	179	39.6
	Science	81	17.9
	Business	62	13.7
	Medicine and Health	60	13.3
	Engineering and Architecture	18	4.0
	Nursing and Midwifery	18	4.0
	Law	17	3.8
	Food and Nutritional Sciences	10	2.2

### Thematic analysis

**Fig 2:**
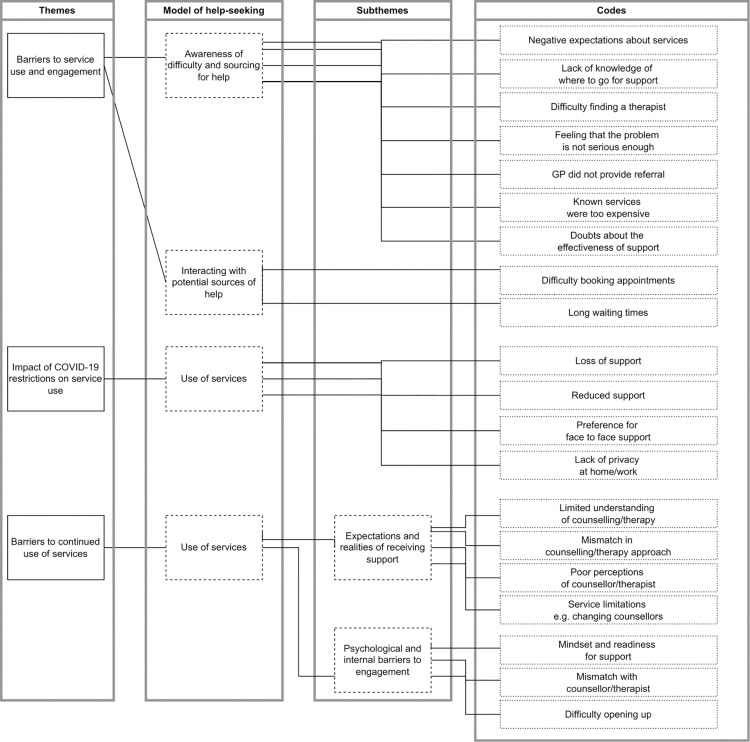
Thematic Map (This image was created by OO).

The thematic analysis of the data led to development of three main themes: “barriers to service use and engagement”, “impact of COVID-19 restrictions on service use”, and “barriers to continued use of support”. The themes represent barriers that students encountered at different stages of their help-seeking journeys. Further, the theme, “impact of COVID-19 restrictions on service use”, represents transient barriers that occurred due to the pandemic. Fig 2 provides a summary of the themes/codes and their relation to the theoretical model.

### Barriers to service use and engagement

This theme outlines the barriers that students face upon recognising their need for support, and while attempting to gain entry into a service. This theme aligns with the model of help-seeking, specifically, awareness of difficulties and sourcing of help, and interacting with services. This theme focuses on challenges faced before attending a service.

Participants described the challenges they faced after recognising their need for mental health support. Some participants noted their expectations of support services had prevented them from seeking support. They mentioned that they had heard that waiting lists were too long and that the service was underfunded or oversubscribed with limited ongoing support. In addition, participants noted that they doubted the effectiveness of support or felt that their issues were not severe enough to receive support. In one instance, a participant reported not receiving a referral to a mental health support service due to their GP’s lack of belief in the efficacy of such services.

Participants mentioned that even though they wanted to access support, they did not know where to go to get support, how to access it, how long any potential wait would be and what to expect from a service. This lack of awareness was a barrier to their use of services. Participants who searched for therapists mentioned experiencing difficulties navigating the websites. They described the search process as time-consuming and challenging. Some participants noted that the cost of counselling/therapy sessions made support inaccessible. One participant noted that subsidised support was equally inaccessible to them, for example:

*I am an international student spending €40k on tuition fees alone per year, so I cannot afford many things here (education comes at a big cost to my lifestyle for me). Even €20 for each counselling session is expensive for me (especially with the exchange rate to my home currency). [P110]*

This indicates that international students may struggle to pay for mental health support services even when they are subsidized, and that their experiences of paying for support may differ from those faced by Irish/EU students. Participants who identified suitable services faced difficulties while attempting to connect to such services. They noted that the process of booking an appointment was complicated, and suggested that an online booking system would make it more straightforward and accessible:

*.. make it easier for people to make bookings. even an online booking system, like the ones restaurants have for making reservations, because if someone’s depressed and see that they have to email someone all this information about who they are and their schedule, its going to demotivate them from seeking help there because if someone is severely depressed, they have very little energy and motivation. [P120]*

The expectation of ease of access is set by other services which are more technologically driven, for example, restaurants. The majority of the survey participants are young adults, who are referred to as digital natives, therefore, they expect mental health services to be better tailored to their needs. Further, participants mentioned being on waiting lists for several weeks and up to 18 months. In other cases, they reported that they had heard nothing back from a service:

*It took a lot of courage to even consider counselling in the first place and so when I made the step to get help but received non[e], it was very debilitating. [P227]*

This indicates that there is often an emotional barrier to seeking support, and at this time, this participant stated that they were questioning the seriousness of their difficulties. Unfortunately, a lack of response from the service confirmed those feelings.

This theme suggests that participants experienced several structural and psychological barriers in the first two stages of their help-seeking journeys i.e. when they had become aware of their difficulties, were considering various sources of help, and when they were interacting with services. These barriers prevented or delayed their use of services.

### Impact of COVID-19 restrictions on service use

This theme represents contextual barriers that arose as a result of the COVID-19 restrictions. Participants noted that the restrictions led to the loss or reduced access to mental health support. In some cases, this loss of support was due to a preference for face-to-face counselling/therapy which was not available at the time. The following comment exemplifies this case:

*due to [the] pandemic face to face sessions were changed to a five-minute phone call and put on medication instead of trying to work through issues [P225]*

Here, the participant describes how the pandemic led to a significant change in the level of support offered by a mental health support service. They also indicate that the treatment provided was not what they wanted. In addition, some participants who had attended online sessions felt that they were not as effective as face-to-face counselling/therapy. Others noted that they didn’t have the level of privacy needed when attending online sessions either at home or at work:

*It was accessible however less efficient than face to face interaction. Harder to become personal over the phone especially when you dont live alone and someone might have caught some of the conversation. [P358]*

Despite attending counselling/therapy sessions, the participant indicates that a lack of privacy hindered their engagement while using a service. The lack of privacy during the pandemic was brought on by travel restrictions and stay-at-home orders.

This theme outlines the barriers participants faced in the third stage of their help-seeking journeys i.e. while using services. These barriers were transient and arose due to the COVID-19 restrictions.

### Barriers to continued use of services

This theme captures barriers faced once a student has gained entry into a service i.e. attended one or more sessions. This theme has two subthemes: expectations and realities of receiving support, and psychological and internal barriers to engagement.

#### Expectations and realities of receiving support.

This subtheme captures barriers related to the discrepancies between participants’ expectations of support and the realities of receiving support.

Participants noted that they had a limited understanding of counselling/therapy and what a session typically entailed. As a result, their attendance at a counselling/therapy session did not match their expectations:

*Perhaps I went in with a closed mind but I didn’t get the crying self help that I know some people get - based on the tissues in the bin...I just didn’t get the wow factor [P408]*

This indicates that an expectation of an emotional response to attending counselling/therapy was developed while in the room with the support person. This expectation led the participant to perceive the session as ineffective.

Participants noted a mismatch between their expectations of counselling/therapy and the approach used by their counsellor/therapist. Some participants felt that the counselling/therapy approach applied was not suitable for their needs. In some cases, participants felt that there was a prioritization of medication over therapy. The following comment exemplifies this case:

*The counsellor was nice but I think CBT was the wrong therapy for me. [P111]*

Many participants felt that the approaches applied by a counsellor/therapist were unsuitable for them, and in many cases this led them to stop their use of a service. Further, participants described their perceptions of their counsellor/therapist. Some felt that they were judgemental, hasty and didn’t take their issues seriously. The following comment exemplifies this case:

*The advice offered was totally generic (ie ’go take a bath/do meditation/ try mindfulness’ in response to suicidal ideation, crippling anxiety and compul[s]ions...) I was made to feel like I was wasting their time. [P442]*

The feedback received after divulging such sensitive information made this participant feel like their issues were not taken seriously. They mention that it “put [them] off seeking help again for years and made [them] feel like [their] problems weren’t big enough” [P442].

Participants highlighted service limitations that did not align with their expectations of support. Examples of this include services where counsellors/therapists were changed from one session to the next, where there were a limited number of sessions or students were discharged when they turned 18 years old. In particular, one participant recalled that following a traumatic event, they had received support one hour every two weeks for a total of four sessions. They described the impact of this level of support on their mental health:

*Instead of having access to frequent therapy, I turned to alcohol and drugs to suppress the feelings distracting myself from what was really going on. I bottle everything up and I have so much anger in me that I never got the chance to get out through talking. [P404]*

This quote illustrates the implication that poor access to treatment can have on a student’s wellbeing. It also highlights that barriers exist in the third stage of help-seeking i.e. after a student has begun their use of services.

This subtheme implies that participants’ expectations of support did not align with the support they received.

#### Psychological and relational barriers to engagement.

This subtheme captures barriers that stem from participants’ internal processes and hinder their ability to fully engage in a service. Participants described how a lack of readiness for support limited the effectiveness of support they had received. Some participants mentioned that they were not in the right mindset to attend counselling and it was not their decision to get counselling:

*I think the reason I found it unhelpful was due to my reluctance to actually engage and give it a go. [P277]*

Some participants felt that they did not “click” with their counsellor/therapist and as a result, had stopped making appointments. Others mentioned that they found it hard to open up:

*I entered the service following a severe panic attack. the service helped me somewhat however I found it hard to open up fully and rarely spoke about my worse issues [P70]*

Although this participant had a willingness to attend a service, they found it difficult to express and disclose their issue to their chosen form of support. This limited the effectiveness of the support they received.

This subtheme implies that a lack of readiness for support and relational barriers limited participants’ engagement in the third stage of their help-seeking journeys i.e. while using a service.

## Discussion

### Key findings

This study explored students’ experiences accessing mental health support in Ireland. To our knowledge, this is the only recent study detailing these experiences in Ireland. Participants in this study describe the barriers they faced upon recognizing their need for support while interacting with and using services, these barriers are underpinned by the model of help-seeking presented in this paper. This model is informed by our data, Rickwood’s model of help-seeking [24] and Andersen’s health service delivery framework [25]. In the first and second stages of the help-seeking model, *awareness of difficulty and sourcing for help* and *interacting with services*, participants experienced barriers that delayed or prevented their use of services. In the third stage of the model, during their *use of services*, participants experienced barriers which led to a discontinuation of that service, and in other cases, it led to a delay in future help-seeking sometimes spanning years.

### Relation to existing literature

Previous research has documented a lack of awareness of services (including how to access and what to expect from services) [26,27], cost [8,27], doubts about the seriousness of symptoms [26,27], and doubts about the effectiveness of support [27] as barriers to seeking mental health support following the recognition of symptoms. In this study, we found that students who knew where to go for support sometimes did not know how to access support, how long the waiting times would be or what to expect in a service. This was a barrier to accessing support. Efforts to provide subsidized support to students are recognized, however, we found that these services were still out of reach for students with limited means, e.g. international students paying high fees. Long waiting lists are a well-documented barrier that exists when young adults are interacting with services [6,28,29]. However, in this study, participants mentioned never hearing back from support services.

Participants who had been using services prior to the COVID-19 pandemic describe their experiences after the restrictions were put in place. Our findings are consistent with the mixed-methods survey by YoungMinds organisation in the UK [15–17]. They found that 28.5% of young adults had lost access to mental health support and 73% were receiving virtual support [15–17]. Some participants found virtual support challenging because of a lack of privacy at home, a preference for face-to-face support or the perception that virtual appointments were less effective than face-to-face support [15–17]. Similarly, they found that appointments had been shortened as a result of the COVID-19 pandemic[15–17].

In this study, participants described barriers they faced while using services. Similar to previous research, students reported being unsatisfied with the approach taken by a support person e.g. prioritisation of medication over therapy [5,6,27,28] and frustrated by service limitations e.g. changing counsellors or limited number of sessions [6,8,26,29]. In the present study, many participants describe being unsatisfied with the approach taken by a counsellor/therapist, which, in some cases, led them to stop their attendance at a service. However, in the case where a participant decided to keep attending, they mentioned feeling frustrated or let down. Additionally, participants noted that limited/infrequent appointments were ineffective, and in one case, this had led to substance abuse. Finally, participants who had negative experiences with a counsellor/therapist noted that it deterred them from seeking help for several years. This finding is consistent with existing research which suggests that negative experiences can affect future help-seeking for an individual and their social network (i.e. friends and peers) [24,30–32].

Finally, research indicates that a lack of emotional competence i.e., the ability to express one’s difficulties can be a barrier to help-seeking among young adults [24]. The present study found that a lack of emotional competence can persist while using services and affect students’ engagement with the support person, therefore, limiting the effectiveness of support received.

### Implications for research and practice

Researchers investigating barriers to students’ use of mental health support services propose anti-stigma campaigns [33,34], raising awareness of services [34], and online mental health programs [35] as ways to encourage help-seeking in this population. However, our findings indicate that barriers encountered following a decision to seek help are quite significant and can deter help-seeking for many years. Consequently, it is important to go beyond awareness campaigns to teaching students how to access support, and what to expect from services. This falls under the definition of the term “Mental health literacy” [36] which is one of the facilitators of help-seeking [24].

Young adults are generally referred to as digital natives but our findings indicate that the process of gaining entrance into a service (e.g. searching a website or booking an appointment) is not well-tailored to this population. These findings resonate with previous research in which participants describe a long intake process involving filling several forms and sending multiple emails, leading to delays in accessing the needed support [26,29]. Mental health support services have to apply technology in a way that is usable and accessible to this population. Despite being digital natives, not all young adults/students desire to participate in virtual appointments, either because of a personal preference or a lack of privacy to attend appointments. Online counselling/therapy should not be promoted as a one-size-fits-all for this population, as it may be a barrier to using these support services. This is especially important in light of plans to further integrate telehealth consultations into the Irish healthcare system [37].

Similar to previous research, many participants reported being unsatisfied with the approach taken by a counsellor/therapist [5,6,27,28]. This indicates a need to provide patient-centred care to students i.e. taking into account their needs concerning the kind of treatment they would like. Previous research suggests that this approach provides a better therapeutic relationship, and can lead to better outcomes for the patient [38,39].

We recognize that services are sometimes underfunded leading to long waiting lists, limited/infrequent sessions, or changing counsellors. Nonetheless, it is important to inform students of service limitations and provide updates to those on waiting lists. Our findings highlight that lack of communication during the waiting period was a barrier to the use of services. Further, in this study, we see the ramifications of poor access to treatment on students’ wellbeing. Therefore, it is important that when students reach out for support, they are provided with appropriate support or are signposted to services that can help them. Previous research recommends having a network of services within and outside the university to encourage good referral practices and to provide students with multiple avenues for seeking support [40,41].

We have explored the student perspectives in seeking mental health support in Ireland. Future research should consider the perspective of other stakeholders including GPs, counsellors, therapists and support services. This will help ascertain how service provision can be improved for this population.

### Strengths and limitations

A strength of this study is a comprehensive examination of the barriers students face while accessing mental health support services in Ireland. The generalizability of our findings may be limited by the use of a single institution as a case study. While our findings resonate with previous research in many areas, future research should conduct a national survey, to account for the broad range of barriers students face when accessing mental health support services in Ireland.

A strength of this study is that participants were representative of all schools at the University and unlike previous research, postgraduate students were not excluded from this study. These findings may be limited as most of the participants were female. It should be noted that at the time of the survey completion, 60% of the registered students at the University were female while 39% were male. Nonetheless, future research should endeavour to include more male participants to ensure that their experiences are well reflected in the findings. The analysis in this study is focused on the totality of barriers faced by students accessing support. There is a benefit in exploring how these experiences differ according to age, gender or nationality. Future research should explore how these factors affect access to support. Our findings should be interpreted in light of these limitations.

## Conclusion

In this study, we explored the students’ experiences while accessing mental health support. Past research has focused on barriers to help-seeking among students, whereas this research highlights the need for a focus on the experiences of students who access mental health support. We address this gap in the literature by exploring what happens between a student’s decision to seek help and their regular use of a mental health support service. Our findings suggest that some students are stuck in the journey of accessing mental health support services. Future research should engage students and relevant stakeholders in in-depth qualitative studies to further explore barriers faced when receiving/providing support to this population. Stakeholder engagement could lead to the design of solutions that can help address these barriers.

**Abbreviations**

GP - General Practioner

UCC - University College Cork

DSS - Disability Support Service

## Supporting information

S1 Appendix Survey questions. (PDF)

S2 Appendix Rationale for help-seeking model. (PDF)
