# Supplementary material for: Students’ attitudes and experiences toward mental health support services in Ireland: A qualitative study
Source: PLoS One. 2025 Aug 21;20(8):e0329905. doi: 10.1371/journal.pone.0329905 (PMC12370073; doi:10.1371/journal.pone.0329905)
Supplement: S1 Appendix — (PDF) [file pone.0329905.s001.pdf]

S1 Appendix

1. **Experiences with support during COVID-19 pandemic**
   - Yes/No: Have you experienced significant problems in the past year (March 2020 - April 2021), and would you have benefited from professional help (e.g., counsellors, psychiatrists, GPs)?
   - If "Yes": What forms of support did you access during this period? Please provide open-ended responses describing your experiences with the support you received during the COVID-19 pandemic. For instance, your experience reaching out for these supports, how helpful/unhelpful the support was and whether you would continue using that form of support.
2. **Experiences of support prior to the COVID-19 pandemic**
   - Yes/No: Have you **ever** used the UCC counselling service to receive mental health support?
   - Open-ended: If "Yes", Please provide open-ended responses describing your experiences with these services, including: how you learned about the service, the process of getting an appointment, and the benefits and shortcomings of the service.
   - Yes/No: Have you **ever** used the other on-campus services to receive mental health support?
   - Open-ended: If "Yes", Please provide open-ended responses describing your experiences with these services, including: how you learned about the service, the process of getting an appointment, and the benefits and shortcomings of the service.
   - Yes/No: Have you **ever** used external services (i.e. services outside the university) to receive mental health support?
   - Open-ended: If "Yes", Please provide open-ended responses describing your experiences with these services, including: how you learned about the service, the process of getting an appointment, and the benefits and shortcomings of the service.
3. **Suggestions for the improvement of mental health support**
   - Open-ended: Do you have any thoughts about the kind of mental health resources or support that students need? How might mental health support be improved for students? Any other comments you would like to add?

Personal experiences detailing the use of mental health support services were included in our analysis.
