# Supplementary material for: Students’ attitudes and experiences toward mental health support services in Ireland: A qualitative study
Source: PLoS One. 2025 Aug 21;20(8):e0329905. doi: 10.1371/journal.pone.0329905 (PMC12370073; doi:10.1371/journal.pone.0329905)
Supplement: S2 Appendix — (PDF) [file pone.0329905.s002.pdf]

S2 Appendix

Following the initial coding of the data, the first author explored the literature on help-seeking and health service utilisation, which led to two frameworks that would help explain the data, namely, Rickwood's help-seeking model [1] and Andersen's health service delivery framework [2]. Rickwood et al. [1] define help-seeking as the process of seeking out help for a personal problem using social relationships. These relationships could fall into two categories: formal (teachers, counsellors, psychologists, etc) or informal (family or friends). They proposed that help-seeking occurs in four stages. In the first stage, a person becomes aware that they have a problem that requires support. In the second stage, they can express this problem in words to a person they are comfortable with. The support must be available and accessible. Finally, they must be willing to disclose their issue to their chosen form of support. Rickwood's model of help-seeking fails to consider the process of finding and shortlisting appropriate services. It also overlooks what happens after a young person begins their use of a service. Additionally, the model does not address external barriers, such as availability and costs [3]. Finally, it does not account for changes in help-seeking over time [4]. Nonetheless, it has useful concepts which help in understanding a student's help-seeking journey and has been applied in other studies to conceptualize help-seeking among young adults [3, 5, 6].
Andersen's health service delivery framework [2], has been applied extensively towards the understanding of health service utilisation in various contexts including mental health (in the general population [7–9] and student population [10]), general health care [8, 9] and maternal care [11]. Key concepts in this framework are access, continuity, and satisfaction. In this model, access is defined as "actual use of personal health services and everything that facilitates or impedes their use" [12, p. 3]. The authors distinguish between initial entry into the system and the continued use of services, which is vital for producing improved health outcomes [2, 12]. Finally, satisfaction is defined as the patient's perception of the quality of care they received, which subsequently affects future utilization of services [2].
The model posits that barriers to access exist within the individual and the healthcare delivery system [2]. For instance, beliefs and values about receiving support, income, insurance coverage, and perceived level of need can impact an individual's use of support [13]. Additionally, characteristics of the healthcare delivery system, such as the number of available health professionals, waiting times, and the clinically evaluated needs of patients, can influence access to care [13]. The characteristics of this model align well with the research question for this study, which is the determination of barriers to access and continued use of mental health support services.
The model of help-seeking presented in this paper is based on knowledge of our data, Rickwood's model of help-seeking [1] and Andersen's health service delivery framework [2]. S2 Table 1 shows the mapping of these theories and our data to the model of help-seeking. It is important to note that this list is not exhaustive but focuses on mapping key concepts that informed the model.

The first stage of the help-seeking model, *awareness of problems and sourcing for help* is informed by the three sources as shown in S2 Table 1. In this stage, a student is aware that they have a difficulty with which they require some help and they are considering where to go for support. Knowledge of where to go for support, and how to search for support may hinder or facilitate their progression into the next stage of the help-seeking journey (based on our data). In addition, incorrect appraisal of symptoms can hinder progression in the help-seeking process (as per Rickwood's model). Further, the ability to express the difficulty in a way that can be understood by others is an important step in the help-seeking process (as per Rickwood's model). Expression can be exercised by

Table 1. Mapping of theories and data to model of help-seeking

**Model of help-seeking**	**Data**	**Rickwood's help-seeking model**	**Andersen's health service delivery framework**
Awareness of problems and sourcing for help	- Knowledge of services and how to search for services<br>- Search for services (e.g. web search, asking friends for recommendations)	- Appraisal of symptoms<br>- Expression of the need for support (e.g. asking a friend)	- Perceived need for support<br>- Beliefs about support
Interacting with potential sources	- Searching for the right service (e.g. sending emails, checking websites)<br>- Booking appointments	- Knowledge of available and accessible support	- Waiting times<br>- Number of professionals in service<br>- Clinically evaluated need<br>- Income/Insurance coverage
Use of services	- Numerous entries and exit from services	- Willingness to disclose difficulty to chosen form of support	- Satisfaction<br>- Continuity<br>- Initial entry into service

asking a friend for recommendations of potential mental health support services or by performing an online search for suitable support [5]. Finally, the perceived need for support and beliefs about support can influence the progression of the help-seeking journey (as per Andersen's framework). In the second stage of the help-seeking model, *Interacting with potential sources*, a student is actively interacting with potential services to select one or more services they can use. Rickwood's model assumes that support is readily available and accessible. However, students might encounter difficulty navigating service websites or a protracted appointment booking process (based on our data). Finally, entry into a service may be affected by waiting times, number of professionals in a service, clinically evaluated need, income and insurance coverage (as per Andersen's framework). In the third stage of the help-seeking model, *Use of services*, a student is actively using services to obtain mental health support. Upon gaining entry into the service, their continued use of the service is based on their satisfaction with the service (as per Andersen's framework). They may use multiple services before finding appropriate support (based on our data). Finally, their engagement in a service may depend on their willingness to disclose their difficulties to support persons (as per Rickwood's model).

References

1. Rickwood D, Deane FP, Wilson CJ, Ciarrochi J. Young people's help-seeking for mental health problems. Australian e-Journal for the Advancement of Mental Health. 2005;4(3):218–251. doi:10.5172/jamh.4.3.218.

2. Aday LA, Andersen R. A Framework for the Study of Access to Medical Care. Health Services Research. 1974;9:208–220.

3. Kauer S, Buhagiar K, Sanci L. Facilitating mental health help seeking in young adults: the underlying theory and development of an online navigation tool. Advances in Mental Health. 2017;15(1):71–87. doi:10.1080/18387357.2016.1237856.

4. Liu J, Zhang Y. Understanding and Facilitating Mental Health Help-Seeking of Young Adults: A Socio-technical Ecosystem Framework; 2024. Available from: `http://arxiv.org/abs/2401.08994`.

5. Pretorius C, McCashin D, Kavanagh N, Coyle D. Searching for Mental Health: A Mixed-Methods Study of Young People's Online Help-seeking. In: Proceedings of the 2020 CHI Conference on Human Factors in Computing Systems. Honolulu HI USA: ACM; 2020. p. 1–13. Available from: `https://dl.acm.org/doi/10.1145/3313831.3376328`.
6. Collin PJ, Metcalf AT, Stephens-Reicher JC, Blanchard ME, Herrman HE, Rahilly K, et al. ReachOut.com: The role of an online service for promoting help-seeking in young people. Advances in Mental Health. 2011;10(1):39–51. doi:10.5172/jamh.2011.10.1.39.
7. Fortin M, Bamvita JM, Fleury MJ. Patient satisfaction with mental health services based on Andersen's Behavioral Model. The Canadian Journal of Psychiatry. 2018;63(2):103–114. doi:10.1177/0706743717737030.
8. Lederle M, Tempes J, Bitzer EM. Application of Andersen's behavioural model of health services use: a scoping review with a focus on qualitative health services research. BMJ Open. 2021;11(5):e045018. doi:10.1136/bmjopen-2020-045018.
9. Babitsch B, Gohl D, von Lengerke T. Re-revisiting Andersen's Behavioral Model of Health Services Use: a systematic review of studies from 1998–2011. GMS Psycho-Social-Medicine. 2012;9:Doc11. doi:10.3205/psm000089.
10. Pilar MR, Cunningham-Williams RM, Williams Woodson SLL. Does the Andersen Behavioral Model of Health Services Use predict college students' use of on-campus mental health services? Journal of American College Health. 2020;68(6):631–643. doi:10.1080/07448481.2019.1583665.
11. Tolera H, Gebre-Egziabher T, Kloos H. Using Andersen's behavioral model of health care utilization in a decentralized program to examine the use of antenatal care in rural western Ethiopia. PLOS ONE. 2020;15(1):e0228282. doi:10.1371/journal.pone.0228282.
12. Andersen RM, Davidson PL. Improving Access to Care in America: Individual and Contextual Indicators. In: Changing the U.S. health care system: Key issues in health services policy and management, 3rd ed. San Francisco, CA, US: Jossey-Bass; 2007. p. 3–31.
13. Andersen R, Newman JF. Societal and Individual Determinants of Medical Care Utilization in the United States. The Milbank Quarterly. 2005;83(4):10.1111/j.1468–0009.2005.00428.x. doi:10.1111/j.1468-0009.2005.00428.x.
